# COVID-19 and obesity: the meeting of two pandemics

**DOI:** 10.20945/2359-3997000000318

**Published:** 2020-12-15

**Authors:** Simone Cristina Soares Brandão, Emmanuelle Tenório Albuquerque Madruga Godoi, Lúcia Helena de Oliveira Cordeiro, Camila Silva Bezerra, Júlia de Oliveira Xavier Ramos, Gustavo Freitas Alves de Arruda, Esdras Marques Lins

**Affiliations:** 1 Universidade Federal de Pernambuco Departamento de Clínica Médica Recife PE Brasil Departamento de Clínica Médica, Universidade Federal de Pernambuco (UFPE), Recife, PE, Brasil; 2 Universidade Federal de Pernambuco Recife PE Brasil Programa de Pós-Graduação em Cirurgia, Universidade Federal de Pernambuco, Recife, PE, Brasil; 3 Universidade Federal de Pernambuco Faculdade de Medicina Recife PE Brasil Faculdade de Medicina da Universidade Federal de Pernambuco, Recife, PE, Brasil

**Keywords:** COVID-19, obesity, pandemics, insulin resistance, inflammation

## Abstract

COVID-19 and obesity are two pandemic diseases that the world is currently facing. Both activate the immune system and mediate inflammation. A sequence of disease phases in patients with severe COVID-19 results in a cytokine storm, which amplifies the subclinical inflammation that already exists in patients with obesity. Pro-inflammatory cytokines and chemotactic factors increase insulin resistance in obesity. Therefore, a greater systemic inflammatory response is establishe, along with an increased risk of thrombotic phenomena and hyperglycemic conditions. These changes further impair pulmonary, cardiac, hepatic, and renal functions, in addition to hindering glycemic control in people with diabetes and pre-diabetes. This review explains the pathophysiological mechanisms of these two pandemic diseases, provides a deeper understanding of this harmful interaction and lists possible therapeutic strategies for this risk group.

## INTRODUCTION

COVID-19 (COrona VIrus Disease 2019) is caused by the Severe Acute Respiratory Syndrome Coronavirus 2 (SARS-CoV-2), and arises from the same family of other coronaviruses responsible for acute respiratory distress syndrome (ARDS), such as SARS-CoV and MERS-CoV (1). It was initially described as a respiratory tropism virus, and the occurrence of extrapulmonary manifestations has been noted. Cardiac, vascular, renal, gastrointestinal, cutaneous, and neurological complications are among the reported features (2,3).

The disease started spreading globally from December 2019, being recognized as a pandemic on March 11, 2020 (4). The first case in Brazil was reported on February 25, and since then the pandemic has spread rapidly. Most of the information about COVID-19 in Brazil was calculated from data provided by several government projects and organizations. As of October 12^th^, 2020, there were more than 5 million confirmed cases. The number of deaths at the time of this writing was above 150,000. The lethality rate in Brazil is 2.96, which is higher than the lethality rate in other Latin American countries such as Argentina (2.6%), Uruguay (2.23%), and Paraguay (2.1%) (5–7).

Older adults and people of any age with certain underlying medical conditions (such as type II diabetes mellitus and serious heart dysfunctions) have been identified as higher risk groups for more severe illness (8). Obesity also plays a leading role over other highly correlated risk factors. According to a study from the Kaiser Permanente in Southern California (a large integrated healthcare organization), there is an association between obesity and risk for death. This risk was most striking among those aged 60 years or younger, and in men (9).

According to the World Health Organization, obesity is also a pandemic disease (10). The prevalence of obesity has tripled since 1975. More than 1.9 billion adults were overweight in 2016, of which 650 million were obese, while another 340 million among children and adolescents aged 5 to 19 years were overweight or obese (11). The prevalence of obesity in Brazil has increased 67.8% in 13 years, from 11.8% of the population in 2006 to 19.8% in 2018 (12).

Many case series have been published around the world reporting the association of body mass index (BMI) with severe cases of COVID-19 (13–16). BMI ≥ 30 kg/m^2^ was present in 47.5% of patients with severe COVID-19 in a French center, regardless of age, diabetes, or hypertension (13). Gao and cols. described a 12% increase in the risk of severe COVID-19 for each unit of increase in BMI (14). In the United States, where 40% of adults are obese, Petrilli and cols. conducted a cross-sectional analysis of 4103 patients with laboratory confirmation for SARS-COV-2, concluding that BMI > 40 kg/m^2^ was a strong predictor of hospitalization with an odds ratio (OR) of 6.2 (15). Furthermore, higher in-hospital mortality was independently associated with BMI ≥ 35 kg/m^2^ with an OR of 3.78, as well as between male gender and aging (16). A harmful inverse correlation was observed in retrospective analyses between BMI and age, in which obesity was described as a risk factor in individuals under 60 years of age (17,18).

The possible links between the severity of COVID-19 and obesity are intriguing to the medical and scientific community. This review aims to explain the possible pathophysiological mechanisms of these two pandemics, providing a better understanding of this harmful interaction and a rational therapeutic strategy for this risk group.

## COVID-19 INFECTION MECHANISM

SARS-CoV-2 is a simple RNA beta-coronavirus with 5.1-day median incubation infection period and a basic reproduction number (an indicator of the disease transmissibility) of 3.28 (R0=3.28). It is mainly transmitted through aerosol, droplets, and fomites. The potential mechanism of SARS-CoV-2 invasion is receptor-mediated endocytosis (19).

SARS-CoV-2 requires angiotensin-converting enzyme 2 (ACE2) receptors as well as transmembrane protease serine 2 (TMPRSS2) for entry into epithelial cells (20,21). The Spike (S) protein of the coronavirus uses the ACE2 from the human host, expressed in alveolar epithelial cells via AT1 receptors, and other tissues such as the heart, the endothelium, the kidney, and the pancreas (22).

**Figure 1:**
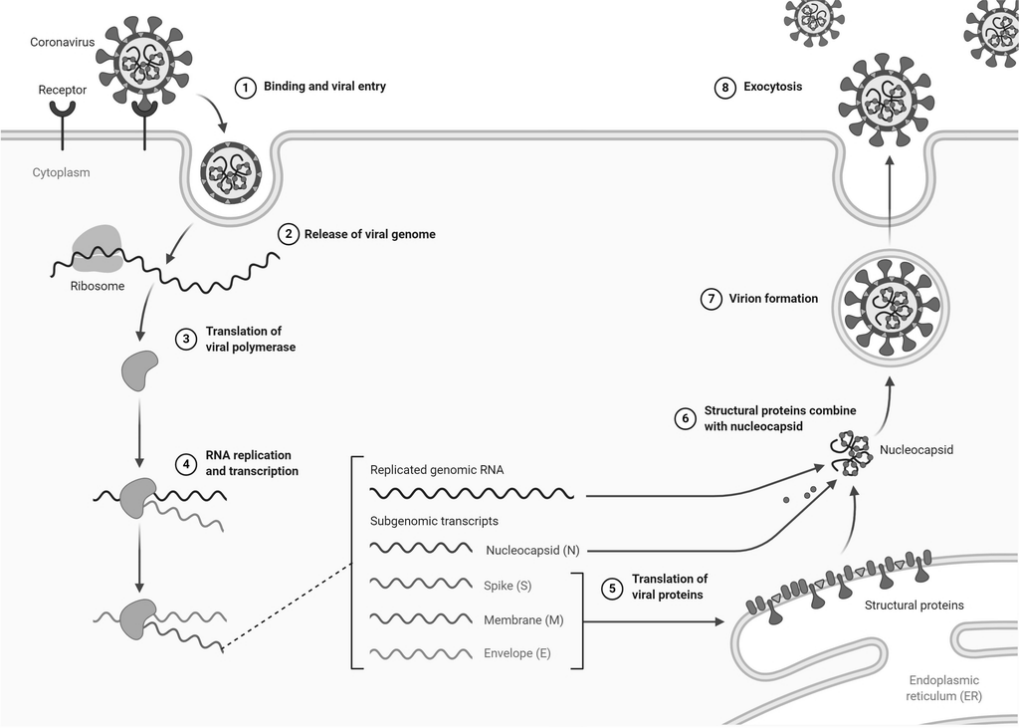
Coronavirus replication cycle. Created by BioRender.com

The S protein is present as a trimer in mature viruses, with three receptor-binding S1 heads sitting on top of a trimeric membrane fusion S2 stalk. SARS-CoV-2 S1 contains a receptor-binding domain (RBD) which specifically recognizes ACE2 as its receptor (23,24). The RBD constantly switches between a standing-up position for receptor binding and a lying-down position for immune evasion. Furthermore, the SARS-CoV-2 S protein needs to be proteolytically activated at the S1/S2 boundary in order to fuse membranes, such that S1 dissociates and S2 undergoes a dramatic structural change. These SARS-CoV entry-activating proteases include cell surface protease TMPRSS2, and lysosomal proteases cathepsins (24,25). The viral translation process takes place as the viral RNA is released into the host cytoplasm, using the host cellular machinery for its own replication and spread (19,26,27) (Figure 1).

Coronaviruses mediate their pathogenic effects by cytocidal and immune-related mechanisms. Cell infection results in cytopathic effects such as cell lysis and apoptosis. The viral complex formed through the replication process leads to disruption of the Golgi complexes, culminating in cell destruction (24,25,28). The immune mechanisms will be elucidated further on.

**Figure 2:**
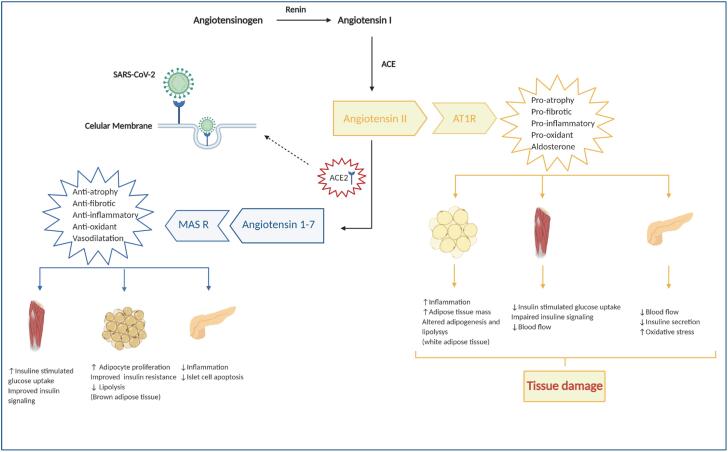
Deregulation of the renin-angiotensin-aldosterone system in COVID-19. The justaglomerular cells of the afferent arterioles of the nephrons release renin which will convert angiotensinogen into angiotensin I. Then, this molecule is converted into angiotensin II (Ang II) through the action of the angiotensin-converting enzyme 1 (ACE), which is complex with its AT1 receptor (AT1R), leading to a pro-inflammatory, pro-oxidative and pro-fibrotic environment. ACE2 converts angiotensin I and Ang II to Ang 1-7, which produces contrary effects to Ang II when it binds to the Mas receptor (MAS R). A reduction or inhibition of ACE2 is observed in COVID-19 due to the binding of SARS-CoV-2 on the cell surface, leading to more severe pro-inflammatory reactions and more extensive tissue damage. Adapted with permission from Brandão SCS and cols. (1,31). Created with Biorender.com.

ACE2 enzymes participate in the angiotensin maturation, a hormone which controls vasoconstriction and blood pressure. It acts by degrading angiotensin 2 (Ang II), the active form of angiotensin, into angiotensin 1-7 (Ang 1-7), serving as a counterpoint to the renin-angiotensin-aldosterone system (RAAS) and protecting the endothelium, heart, lungs, kidneys, and intestines against tissue damage (29) (Figure 2).

Thus, COVID-19 causes an imbalance of RAAS as its entry into cells leads to internalizing ACE2, decreasing its expression on the cell surface. This suppression makes the infected host more susceptible to tissue damage which is stimulated by the vasoconstrictive, pro-oxidative, and pro-inflammatory action of Ang II (29,30).

COVID-19 is classified into three stages according to the clinical presentation of the disease. The viral replication occurs in phase I. Patients may not manifest symptoms or only present flu-like symptoms such as fever, fatigue, coughing, anosmia, odynophagia, and/or even gastrointestinal manifestations. About 80% of patients remain in this phase, with the disease being self-limited and benign. However, more serious manifestations such as ARDS (phase II or hyperinflammatory) may appear in about 15% of those infected, while there is a need for ventilatory support and intensive care (phase III – critically ill patients) in 5% of patients (31). Phase III is characterized by a state of pro-inflammatory hypercytokinemia, called a “cytokine storm” (32).

## IMMUNE RESPONSE AND CYTOKINE STORM

Innate and adaptive immunity is stimulated after the virus enters the cell. The former includes Natural Killer (NK) cells producing several pro-inflammatory cytokines and chemotactic factors such as type I interferons (IFN1), gamma interferon (IFN-γ), tumor necrosis factor-alpha (TNF-α) and interleukins (IL) −1, −6, −8, −12, −15 and −18. On the other hand, the adaptive immune response involves a more specific attack on the virus, recruiting CD4+ and CD8+ T lymphocytes, being responsible for the immune memory (19).

Additionally, in severe COVID-19 there is a dysfunction of NK cells and a reduced IFN1 production, which is the main inducer of the antiviral response (19,29); there is also excessive recruitment and uncontrolled activation of infiltrating pro-inflammatory cells (neutrophils and monocytes-macrophages). McGonagle and cols. suggest that this cytokine storm is caused by the high viremia of SARS-CoV-2 due to a weak immune response at the onset of the disease (32).

The presence of the virus in the pulmonary alveoli and the release of cytokines lead to a loss of epithelial cells and pneumocytes with a subsequent decrease in surfactant, which in turn induces an increase in the alveolar surface tension and the collapse of these structures. As a result, the alveolar cavities are filled with cellular remnants of inflammation and exudate which is aggravated by the increase in cellular permeability caused by Ang II overload (33).

Necropsy studies reveal lymphocytic endotheliitis in the lungs, heart, kidney, and liver, as well as the presence of cellular necrosis, microthrombi, pulmonary intussusceptive angiogenesis, and other pulmonary vascular features in the lungs. In addition, a *postmortem* histology series demonstrated that SARS-CoV-2 may directly act in endothelial cells, with evidence of endothelial and inflammatory cell death. Furthermore, the microthrombotic pulmonary angiopathy related to SARS-CoV-2 may end up in a state of alveolar hypoperfusion (33–36).

The disease progression leads to dyspnea, hypoxia, and classic “ground glass” images on the periphery of the lungs. These manifestations are not specific to COVID-19, as other viral pneumonia (influenza and cytomegalovirus) can also trigger them (34).

The hyperinflammatory phase is marked by laboratory changes such as high levels of C-reactive protein, procalcitonin, ferritin, and D-dimer, with the latter probably being related to the extensive pulmonary microthrombosis (32–34). The increase in circulating macrophages and the hypersensitivity of CD4+ T cells increase the IL and TNF-α levels, as well as the granulocyte-macrophage colony-stimulating factors, which leads to calling for even more leukocytes, thus maintaining the hypercytokinemia state (31,34).

This profile is similar to Macrophage Activation Syndrome (MAS), in which an excess of cytokines occurs due to inadequate macrophage elimination, featuring persistent fever, cytopenias, liver dysfunction, and disseminated intravascular coagulation. However, unlike in MAS, respiratory failure and atypical pneumonia are more common in COVID-19, in addition to hypercoagulability, multiple organ dysfunction, and sepsis (32).

All of these systemic repercussions are characteristic of the severe form of COVID-19, and are more prevalent in at-risk populations as described above (37). Thus, it is important to understand the relationship between SARS-CoV-2 infection and obesity.

## OBESITY AND RISK OF SEVERE COVID-19

Overweight and obesity are related to immune system dysfunction, chronic systemic inflammation, insulin resistance, pulmonary, cardiac, and endothelial structural and functional impairment, prothrombotic abnormality, in addition to liver and kidney dysfunction. All of these abnormalities confer a higher risk of developing severe COVID-19 (38).

It is noteworthy that the typical primary increase in the inflammatory response of obesity may be a contributing factor to the hyperinflammatory state observed in severe COVID-19 (39). In addition, adipose tissue was identified as a potential target and a viral reservoir due to increased ACE2 expression (40).

The occurrence of immunological dysfunction with a greater predisposition to infection and mortality from sepsis is described among patients with obesity (41). It is well known that fat accumulation, mainly ectopic accumulation, favors developing inflammation in adipose tissue. This occurs due to an inflammatory state being created with a higher level of inflammatory cytokines, such as IL-6, IL-8, monocyte chemoattractant protein-1 (MCP-1/CCL2), leptin, and plasminogen activator inhibitor-1, and a reduction of adiponectin, an anti-inflammatory adipocytokine. This scenario leads to an increase in recruitment of macrophages (42–44).

Adaptive immunity is also affected in obesity due to a decline in CD4-T cells, as well as an imbalance of CD4-T helper cells. Peripheral counts of CD4 and CD8-T cells are similarly low in patients with COVID-19, which is demonstrated as lymphopenia, a surrogate marker of severe COVID-19, albeit with a higher ratio of pro-inflammatory Th17 cells (39,40,45).

As a consequence, the proportion between pro-inflammatory and anti-inflammatory cytokines becomes unbalanced, promoting endothelial dysfunction and increased arterial stiffness. This state of endothelial dysfunction is characterized by decreased nitric oxide production and increased reactive oxygen species production, amplifying the vascular damage (39).

### Obesity and insulin resistance

Obesity, notably visceral adipose tissue, leads to insulin resistance which results in increased liver production and reduced peripheral glucose uptake, especially in muscle and adipose tissues (45). This also stimulates lipolysis and creates an inflamed and hyperglycemic environment (46).

Adipose tissue is dynamic and interacts with insulin sensitivity and metabolic control, blood pressure status, vascular regulation, and angiogenesis. ACE2 receptors are also expressed in various tissues such as skeletal muscle, endothelium, adipose tissue, and β-pancreatic cells (47).

ACE2 plays an important role in insulin sensitivity through angiotensin 1-7 production (48) (Figure 2). Ang II is believed to reduce the action of glucose transporter 4 membrane receptors on muscle in animal models, decreasing the entry of glucose into the cell. Ang II also alters the adipogenesis, increasing in number and accelerating the differentiation of adipocytes in visceral tissue (49).

SARS-CoV-2 acts deregulating RAAS while using ACE2 receptor as a ligand. The result is an exacerbation of the aforementioned energy metabolism abnormalities in people with obesity and with or without diabetes (50,51).

### Respiratory function in obesity

The lung is an organ that produces inflammatory mediators, and it is not uncommon for patients with obesity to present atopy and bronchial hyperresponsiveness with a greater predisposition to bronchial asthma and pulmonary infections (52).

It is also noteworthy that obesity promotes venous stasis in the lower limbs and chronic venous insufficiency contributing to venous thromboembolism, which provides a greater risk of pulmonary embolism, especially in association with immobility (53).

Patients with obesity are at a higher risk of developing serious pulmonary complications with COVID-19, such as greater chance of requiring oxygen therapy, intubation, and mechanical ventilation (54), in addition to higher in-hospital mortality, occurring in as many as 88.1% of those who required mechanical ventilation (55).

The increased risk of severe COVID-19 in people with obesity is related to a set of structural, inflammatory, macro- and microcirculatory factors, and also to more complex hospital care (31). Ectopic fatty accumulation in the thoracic and diaphragmatic muscles could lead to decreased muscle function, less thoracic expansion, and increased respiratory effort, which, in turn, impairs gas exchange and leads to consequent respiratory dysfunction (52). However, the literature suggests that ectopic accumulation of lipids can appear in cells of the pulmonary alveolus in states of overnutrition, resulting in ultrastructural abnormalities and altered surfactant production (56). Moreover, SARS-CoV-2 may act directly on the endothelium and cause a diffuse endotheliitis with consequent alveolar thrombotic microangiopathy (28,29,57,58).

Another aggravating factor present in patients with obesity is the difficulty to implement the prone position. This life-saving measure in patients with ARDS improves the ventilation/perfusion ratio and arterial oxygenation by using the effects of gravity and repositioning the heart in the chest, improving respiratory system mechanics and alveolar ventilation (59).

### Cardiac function and hypertension in obesity

Obesity produces hemodynamic changes which contribute to developing structural and functional cardiac abnormalities. These changes can cause heart failure (HF), even in the absence of other comorbidities such as hypertension and coronary artery disease (CAD). HF related to severe obesity is called “obesity cardiomyopathy” (37).

Direct and indirect mechanisms are associated with the development of cardiomyopathy due to obesity. Inflammatory mediators produced by adipose tissue are direct mechanisms of cardiac dysfunction and hypertension, while diabetes and CAD are among indirect mechanisms. Inflammation and insulin resistance with hyperinsulinemia are the main baseline changes in obesity which trigger a series of harmful events, culminating in damage to the cardiovascular system (60).

In addition to endocrine factors in people with obesity, hypertension is aggravated by its multifactorial nature with errors in eating habits and genetics (56). Each 5 kg/m^2^ increase in BMI results in an increase of 5 mmHg in systolic blood pressure and 4 mmHg in diastolic blood pressure (61).

Regarding COVID-19, SARS-CoV-2 further exacerbates the deleterious effects of obesity on the cardiovascular system since it acts on ACE2 by deregulating RAAS through direct action on myocardial and endothelial cells, causing inflammation and cell apoptosis. It also leads to a state of systemic hyperinflammation and hypercoagulability. Increased myocardial injury biomarkers such as troponin and brain natriuretic peptide have been described in the severe form of COVID-19, and are markers of higher mortality (40).

Acute cardiac injury is the most reported cardiovascular abnormality in COVID-19, with an average incidence of 8-12% (62). Clinical features of acute myocardial injury are elevated serum troponin level, arrhythmias, and ST-segment elevation and/or depression on the electrocardiogram in the absence of obstructive CAD (63).

Autopsies performed on three COVID-19 patients revealed that the main cardiac findings are cardiomegaly, individual cardiomyocyte injury, lymphocytic epicarditis/pericarditis, and lymphocytic myocarditis. It is noteworthy that all three subjects autopsied were men with obesity (BMIs of 33.8, 51.65, and 35.2 kg/m^2^) and had evidence of chronic heart disease (hypertensive left ventricular hypertrophy, dilated cardiomyopathy, and hypertrophic cardiomyopathy) (64).

### Obesity and its relationship with pre-diabetes and diabetes

Obesity increases the risk of pre-diabetes and diabetes, and all of these morbidities increase susceptibility to hyperinflammation and the cytokine storm syndrome in COVID-19 (65). Guan and cols. showed that approximately 7% of patients with COVID-19 generally had diabetes as a comorbidity. However, the prevalence of diabetes was almost three times higher in patients with severe COVID-19 (16.2%) compared to those with the non-severe disease form (5.7%) (66).

The hyperglycemic environment can interfere with immunity, making the entry of SARS-CoV-2 more efficient and decreasing viral clearance by reducing T cell function. The Dipeptidyl Peptidase-4 (DPP-4) expressed in many tissues deserves some attention in coronavirus infection. The DPP-4 serves as an entrance mechanism for MERS-CoV in human cells, although there is still no evidence for SARS-CoV. However, inhibition of DPP-4 is an important treatment strategy for the treatment of type 2 diabetes and is under investigation (67).

Patients with diabetes have multiple associated comorbidities such as hypertension and high cardiovascular risk (66), as well as non-alcoholic fatty liver disease (NAFLD) (68).

Advanced stages of NAFLD are related to greater insulin resistance, mainly mediated by inflammatory pathways, possibly accelerating progression to cirrhosis in people with pre-diabetes and diabetes. Furthermore, the presence of steatohepatitis is associated with death from cardiovascular disease (69).

Zheng and cols. suggested that obesity and NAFLD may worsen the severity of COVID-19 by gathering data from three reference hospitals. The sample included 214 patients (mean age 47 years and 74% female) with a mean BMI of 22.7 ± 2.1 kg/m^2^ (non-obese group) and 28.3 ± 3.2 kg/m^2^ (obese group). The association of COVID-19 and obesity increased the risk for the severe form of the disease by up to three times, while this risk was six times greater in the presence of NAFLD (68).

Moreover, new-onset diabetes as well as severe diabetes-associated complications such as ketoacidosis and hypernatremia have been described in COVID-19 disease, and are under investigation by the CovDIAB project to address a potential diabetogenic effect of COVID-19 (67,70).

### Obesity and kidney disease

The link between obesity and chronic kidney disease (CKD) can be explained by sharing pathophysiological pathways (such as chronic inflammation, increased oxidative stress, and hyperinsulinemia) and by risk factors and associated diseases (insulin resistance, hypertension, and dyslipidemia). Obesity aggravates hypertension, activating the RAAS and the sympathetic nervous system at the adipocyte level, thus culminating in vasoconstriction, as well as salt and water retention. The association of obesity with other pathologies such as diabetic nephropathy, hypertensive nephrosclerosis, and segmental and focal glomerulosclerosis also amplifies kidney damage (71).

Although the pathogenesis of kidney failure in severe COVID-19 is not yet fully understood, data suggest that complications may be mediated by high concentrations of pro-inflammatory cytokines, including IL-6 and coagulation disorders, which lead to thrombosis. Coagulopathy contributes to acute kidney injury in patients with COVID-19. Reports of hypercoagulability in dialysis filters are described in the disease series. Endotheliitis, angiogenesis, fibrin and microthrombi were also found in an autopsy of a transplanted kidney, possibly causing acute kidney damage in this patient (26,28,72).

## PREVENTIVE AND THERAPEUTIC APPROACHES

There are nearly 135 vaccines against SARS-CoV-2 under development in the world to date, 42 of which are in human clinical trials. However, none of these are authorized for application in large populations, although it is expected that at least one effective vaccine might be licensed by 2021 (73). This will be fundamental for potent control of horizontal transmission, but also future studies may be needed to understand how obesity, hyperglycemia, and hyperinsulinemia might affect their efficacy (74,75).

Patients with obesity have been reported to have impaired development of immunological memory when vaccinated against tetanus, hepatitis A and B, rabies, and influenza (76). Regarding the influenza vaccine, reports show that antibody titers wane rapidly in these individuals during the first-year post-vaccination when compared to non-obese. Obesity may also be related to a higher risk of influenza-like illness as a response, reinforcing the need for including BMI as a variable in trials evaluating vaccine safety and effectiveness (76).

In the meantime, lifestyle changes such as wearing masks, social distancing, and reinforcing personal hygiene are recommended for preventing COVID-19 (74). It is also essential for people with obesity to incorporate behavioral measures such as regular physical exercise and to adopt a balanced diet enriched with fruits and vegetables (75). Diets with higher protein content and lower glycemic index can be effective and should be considered for individuals with diabetes (56,77).

Modest changes in diet may produce benefits to help control the inflammatory response in COVID-19 (75). Clinically achievable weight losses of about 5% or even 10% can improve insulin resistance and liver fibrosis in individuals with obesity, in addition to reducing outcomes like cardiovascular events and mortality (78,79).

However, there is no magical number for this weight loss, nor any data regarding this recommendation and its impact on either infection susceptibility or severity (79). Rather than a “solution” to obesity, these changes may be seen as a “controlled obesity” status, as suggested by Halpern & Mancini, and future COVID-19 studies should consider voluntary weight loss as a potential factor for reduced health risks, even if their BMI is not considered “normal” yet (79). Another concern is that these diet modifications must be guided by professionals, as the pandemic's psychosocial effects on eating behaviors are still unknown, potentially leading to aggravation of eating disorders (80).

Moderate- to vigorous-intensity aerobic exercises are also related to immunological benefits, mostly associated with the release of potent anti-inflammatory cytokines and to decrease of toll-like receptors (TLRs) expression, which are signals for macrophage recruitment and producing of pro-inflammatory cytokines (81). This could be even more helpful for individuals living in a chronic inflammatory status, such as those who have obesity and/or diabetes. Therefore, exercise could enhance long-term response to a vaccine, lower the risk, duration, and severity of the infection, and optimize lung function, avoiding uncontrolled lung inflammation (81).

Furthermore, postponed bariatric surgeries during the pandemic, as well as the follow-up of recently operated patients, might have provoked serious harm for these individuals (70). These surgeries have been related to reduced rates of diabetes, cardiovascular mortality, and cancer, which may also be helpful to avoid severe forms of COVID-19 (82). Hence, metabolic and weight monitoring should be maintained while waiting for the resumption of elective surgeries, and a consensus for prioritizing criteria should be established in order to lessen damages (70).

Regarding potential treatment options for COVID-19, non-pharmacological strategies in the first phase of COVID-19 (asymptomatic or mildly symptomatic individuals) are provisional and effective measures as specific drugs are still not available. Early case identification and isolation, rigorous glucose monitoring, and dose adjustment of antidiabetic drugs might be the best approaches to lessen adverse outcomes such as hypoglycemia, which might occur as a result of possible drug interactions with SARS-CoV-2 pathophysiology (74,83).

Despite this, no hypoglycemic medications except for insulin have had safety studies conducted for diabetes treatment in COVID-19, although some drugs are being tested in ongoing clinical trials (83–85).

Regarding the use of antihypertensive drugs such as ACE inhibitors (ACEi) and angiotensin receptor blockers (ARBs), and despite initial concerns that they could lead to a more aggressive presentation, a recent randomized multicenter trial named BRACE CORONA has shown that stopping these drugs does not reflect clinical benefit for those patients, so they should be maintained if indicated (86,87).

A hyperinflammatory state takes place in COVID-19 phase II with broader pulmonary involvement and hypoxia. The use of corticosteroids has been suggested for patients receiving respiratory support after findings in the RECOVERY study associated a dose of 6 mg/day of dexamethasone with reduced mortality and reduced necessity for mechanical ventilation (88).

Full anticoagulation in confirmed cases or with strong clinical suspicion of venous thromboembolism is already recommended, as well as prophylaxis for hospitalized patients (89). Prophylactic anticoagulation has also been considered at the disease phase I and after hospital discharge, taking into account the hypercoagulable state presented in COVID-19, as well as the anti-inflammatory effect of heparin (90).

Finally, oxygen supplementation measures, the prone position, and intensive care unit admission with ventilatory and hemodynamic support are adopted in phase III. Dialysis and extracorporeal membrane oxygenation may also be necessary in very severe patients (35).

## CONCLUSIONS

COVID-19 and obesity are two pandemics which are capable of provoking fearful results when coinciding. Considering all the aspects discussed in this review, strategies to combat obesity are urgent. Several non-pharmacological and pharmacological measures are part of the therapeutic arsenal of obesity and its complications. Clinical treatment for severe COVID‐19 is still challenging and developing specific treatments is needed. The best treatment while waiting for the vaccine is perhaps one which includes therapies to improve the metabolic and cardiovascular conditions of these patients in order to prevent the worsening of both diseases.
